# Application of Poincare-Mapping of Voiced-Speech Segments for Emotion Sensing

**DOI:** 10.3390/s91209858

**Published:** 2009-12-03

**Authors:** Krzysztof Ślot, Łukasz Bronakowski, Jaroslaw Cichosz, Hyongsuk Kim

**Affiliations:** 1 Institute of Electronics, Technical University of Lodz, Poland, Wolczanska 213/215, 90-924 Lodz, Poland; E-Mails: kslot@p.lodz.pl (K.S.); lukasz.bronakowski@p.lodz.pl (L.B.); jarekcichosz@poczta.onet.pl (J.C.); 2 Division of Electrical, Electronics and Computer Engineering, Chonbuk National University, 561-756 Chonju, Korea

**Keywords:** emotion sensing, feature selection, Poincare-maps, decision-trees

## Abstract

The following paper introduces a group of novel speech-signal descriptors that reflect phoneme-pronunciation variability and that can be considered as potentially useful features for emotion sensing. The proposed group includes a set of statistical parameters of Poincare maps, derived for formant-frequency evolution and energy evolution of voiced-speech segments. Two groups of Poincare-map characteristics were considered in the research: descriptors of sample-scatter, which reflect magnitudes of phone-uttering variations and descriptors of cross-correlations that exist among samples and that evaluate consistency of variations. It has been shown that inclusion of the proposed characteristics into the pool of commonly used speech descriptors, results in a noticeable increase—at the level of 10%—in emotion sensing performance. Standard pattern recognition methodology has been adopted for evaluation of the proposed descriptors, with the assumption that three- or four-dimensional feature spaces can provide sufficient emotion sensing. Binary decision trees have been selected for data classification, as they provide with detailed information on emotion-specific discriminative power of various speech descriptors.

## 1. Introduction

Emotion sensing has become an increasingly important research direction in speech analysis. A successful solution to this challenging problem would enable a wide range of important applications. Correct assessment of the emotional state of an individual could significantly improve quality of emerging, natural-language based human-computer interfaces [1]. It can be applied for monitoring individual's psycho-physiological ability to operate in several demanding work environments or for the assessment of stress or fatigue levels. Emotional speech can provide invaluable clues in forensics. Finally, correct assessment of the emotional state can improve performance of speech and speaker recognition systems (performance of both of the analyses is known to drop sharply if emotional-speech is subject to analysis [2,3]).

Despite a large amount of research that has been done in the field of speech-based emotion recognition [4-7], the problem is still far from a satisfactory solution. The performance of methods that have been proposed so far is rather low, although some of the reported classification rates (at the 70% level for multi-category problems) are comparable to human recognition capabilities. There exist numerous difficulties in emotional-speech analysis. Emotions are expressed through mutual interactions of a variety of processes, originating at various speech-formation levels: generative, articulative and prosodic. These processes have complex and ambiguous representation, dispersed among several speech-signal features. Another fundamental problem of the domain is difficulty in collecting reliable experimental material. There exists no widely accepted methodology for registering and labeling genuine emotional speech. An idea to gather the experimental material by inducing some particular emotional states, is relevant only for some emotions (e.g., anger or boredom) and raises several ethical problems. Therefore, different recognition algorithms are predominantly tested on databases of speech collected from actresses and actors. Unfortunately, such experimental material is not guaranteed to correctly reflect the real emotional processes.

Utterance-based assessment of emotions is a pattern recognition problem. Therefore, the standard pattern recognition methodology, which involves feature space derivation and feature classification, is used to do the task (with the strong emphasis on the first of the two aforementioned elements). A search for novel, appropriately tailored descriptors of emotional speech is a focus of majority of the proposed methods and several features have been identified as important emotion indicators. They can be broadly classified into three major categories [8-10]. The first one is concerned with signal energy representation and includes statistical characteristics of various aspects of signal energy evolution as well as spectral and cepstral parameters of utterances. The second category focuses on pitch and its temporal evolution. Finally, the third category groups a variety of temporal features of a speech signal, such as duration, speaking rate, pause percentage or voiced-speech percentage. Recognition of the produced feature vectors is typically performed using well-established strategies, ranging from vector classification methods, such as Support Vector Machines [11], Neural Networks [12], or k-NN, to vector-sequence classification techniques, such as Hidden Markov Models [10,12,13].

The following paper introduces a set of novel candidates for emotional speech description. These are statistical properties of Poincare Maps that are produced for a set of voiced-speech characteristics. The proposed pool of descriptors is intended to reflect variability in phoneme pronunciation. Assuming that the pronunciation variability is emotion-specific and that it expresses some mechanisms that are different than these involved in production of pitch, energy and temporal components of the speech signal, one can hypothesize that the proposed descriptors could introduce useful, novel information for emotion classification.

To verify this hypothesis, the following feature evaluation methodology is adopted: a broad pool of features that are commonly used in emotion sensing is supplemented with the proposed descriptors and classification-performance driven feature-selection is executed on the produced set. Binary decision trees, where individual emotions are being extracted at consecutive tree-levels, are used as a classification strategy. This approach enables to evaluate emotion sensing performance and, additionally, provides the basis for identification of the most discriminative, emotion-specific feature sets.

Experimental evaluation of the proposed approach has been made for a six category emotion classification problem (joy, anger, boredom, sadness, fear and neutral) and involved databases of emotional speech of two different languages: German [14] and Polish [15]. It has been shown that introduction of Poincare map descriptors significantly (by approximately 10%) improves emotion recognition performance. The majority of the best-performing feature spaces include at least one of the novel descriptors. Also, the resulting emotion recognition rates are very high: for the speaker-independent, six-category classification problem correct classification at the level of 79% for the German and 81% for the Polish database is obtained.

### 2. Vowel Pronunciation Variability Assessment Using Poincare Maps

Speech production is a complex process that can be approached from a nonlinear system dynamics perspective. Speech has been shown to posses several properties of chaotic signals, such as sharply-decreasing autocorrelation function or wide-band energy spectrum [16-22]. Nonlinear system theory provides several tools for the analysis of nonlinear dynamics of speech production. One of them is Poincare-mapping [23], which can be performed for one-dimensional signals by event-synchronized signal sampling. Assuming that pitch is selected to determine consecutive sampling instances $t_{i}^{p}(i=1,2\ldots )$, Poincare-mapping of a signal *x(t)* is the transformation ψ, defined as [24]:

(1) $$x_{i+1}(t_{i+1}^{p}+\Delta t)=\Psi [x_{i}(t_{i}^{p}+\Delta t)]$$

where Δ*t* is some fixed latency. The mapping (1) can be applied to an original speech-signal or to any function defined over this signal. We found, that particularly distinctive, emotion-specific maps are produced for temporal evolution of four speech formant frequencies and for temporal evolution of its total energy.

**Figure 1.**
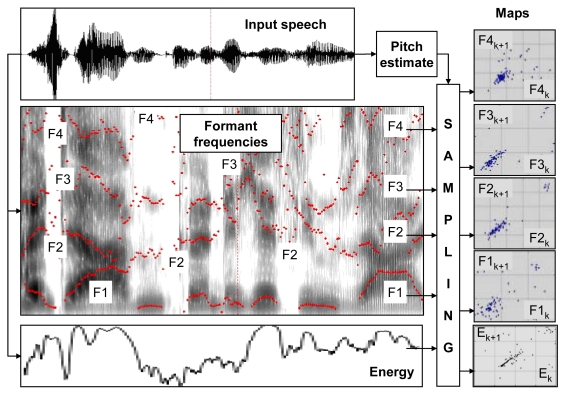
Block diagram of the adopted procedure for Poincare-mapping of the selected speech signal characteristics.

A procedure adopted for derivation of Poincare-Maps, involves detection of voiced speech segments, pitch estimation and extraction of formants. This is followed by pitch-synchronized sampling of formant frequency evolution and total energy evolution, performed for detected voiced-speech intervals (Figure 1). Voiced-speech detection and pitch frequency estimation have been done using the PRAAT software [25]. Of two commonly used ways of Poincare maps visualization: sample-scatter plots (*i.e., x_i_*_+1_ versus *x_i_*) and first-order difference plots (*i.e., x_i_*_+2_ – *x_i_*_+1_ versus *x_i_*_+1_ – *x_i_*) [19], the former is used throughout the paper.

**Figure 2.**
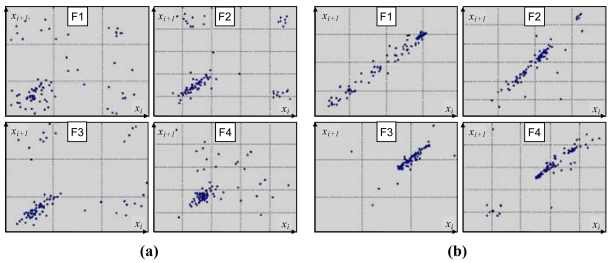
Poincare Maps produced for four formants (F1 through F4) extracted from a sentence uttered with the emotion ‘fear’ (a) and ‘joy’ (b).

**Figure 3.**
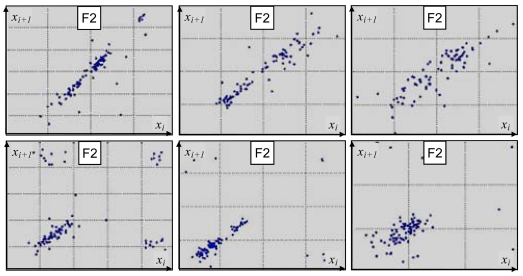
Poincare Maps produced for the second formant evolution for the same sentence uttered by three different speakers with two emotional loads: ‘joy’ (top row) and ‘fear’ (bottom row).

Sample Poincare maps derived for voiced-speech segments of a sentence, uttered with two different emotional loads: ‘fear’ and ‘joy’ (Figure 2), show substantial differences. As it can be seen, a plot is more scattered and less structured for the ‘fear’ class than for the ‘joy’ category, suggesting more chaotic nature of the underlying emotion. Although plots derived for a given emotion vary among different speakers, one can observe noticeable within-class similarities in their overall structure (Figure 3).

To quantify the hypothesized category-dependent appearance of plots, four Poincare-Map descriptors have been proposed. The first two parameters: the determinant and the trace of a co-variance matrix of data points are expected to account for total scatter of samples. The third parameter—the *coefficient of determination, i.e.*, a squared correlation coefficient, defined as:

$$R2={\left(\frac{\mathrm{cov}(x_{i+1},x_{i})}{\sigma (x_{i+1})\sigma (x_{i})}\right)}^{2}$$

where cov(…) stands for co-variance matrix entry and *σ* denotes the standard deviation—is supposed to assess a level of statistical independence between samples. The last descriptor: a *sum of the squares due to error*, defined as a sum of squared differences between samples and their linear regression predictors (*x̃_i_*_+1_ = *αx_i_* + *β*):

$$\text{SSE}(x)=\frac{1}{n}\sum_{k=1}^{n}{\left((ax_{i}^{k}+\beta )-x_{i+1}^{k}\right)}^{2}$$

provides another way of measuring data consistency.

The introduced descriptors have been found to be relatively weakly correlated with features that are commonly used for emotional speech recognition. Sample correlation coefficients between emotional-speech descriptors, evaluated for the databases [14,15], are presented in Table 1. The proposed parameters (determinant—‘det’, coefficient of determination—‘R2’ and sum of the squares due to error—‘SSE’) are confronted with representatives of the three main groups of common speech descriptors: mean pitch—MP, mean energy—ME and normalized utterance duration—ND. The presented results show that processes that underlie phone-pronunciation variability are insufficiently represented among traditionally used speech characteristics. Also, one can expect novel information to be introduced into emotion recognition if feature spaces spanned by commonly-used emotion descriptors are augmented with the proposed parameters.

## 3. Descriptor Evaluation Methodology

The proposed descriptors of voiced speech variability are intended to introduce novel and useful information into emotion sensing process. To verify whether this is actually the case, a pool of features that are commonly used for emotion sensing has been extended with the proposed descriptors and a search for feature subsets, which provide the highest emotion-classification rates, has been performed. We were interested in finding out, whether speech-variability descriptors would appear among the winning feature subsets and in estimating recognition rates that could be attained.

In searching for the most discriminative feature spaces, we assumed the length of a feature vector to be no greater than four. This restriction can be justified since it seems that no more than four sufficiently distinctive processes of emotional speech-production can be identified. These are summarized using characteristics that reflect speech-generation energy, speech-production frequency, temporal events and short-term pronunciation variations. Cross-correlations among features from the same category are significant, so it is rather unlikely to improve recognition performance by extending feature vectors beyond the assumed maximum length. Moreover, restricting a size of a feature vector allows avoiding problems with the curse of dimensionality.

The particular emotions that have been considered in our experiments are: “anger”, “fear”, “sadness”, “boredom”, “joy” and “neutral” (no emotion). Discriminative properties of different feature spaces were evaluated by using emotion classification performance as the criterion function. Binary decision-trees have been adopted as the emotion-classification strategy, since they enable identification of the best-performing features for individual emotions.

### 3.1. Derivation of Feature Spaces

A pool of features that have been used throughout our experiments included elements of the four groups of emotional-speech descriptors. The first group of features comprises statistical parameters pertaining to the speech-production intensity. These are for example, mean or median energy computed over the whole signal, descriptors of local minima and maxima of energy, formant energies, as well as spectral energies evaluated within selected frequency bands. The second group of features addresses processes underlying pitch-generation and comprise its mean, median, range and variance, long-term pitch variations, and descriptors of local pitch landmarks, such as means and standard deviations of local pitch minima and maxima. The third group of adopted features reflects a rhythm and prosody of speech, and includes normalized utterance duration, zero-crossing rate and its statistical descriptors, as well as percentage of pauses and voiced-speech segments. In addition to the presented, commonly used features, we decided to examine also linear, third-order and exponential regression of energy and pitch temporal changes, and to include corresponding coefficients as another group of emotional speech descriptors. Together with the proposed twenty descriptors of voiced-speech variability (four descriptors per each of the five processes that were subject to Poincare mapping), the pool of features considered for emotion recognition comprised a total of 146 elements.

**Figure 4.**
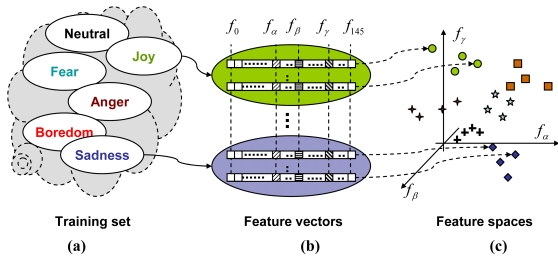
Training set with labeled emotional sentences (a), 146-element feature vectors derived for each sentence (b) and sample three-dimensional feature space, with training-set samples from all of the categories (c).

All examples available for experiments have been split into training and test sets. For each training-set sentence, all of the considered 146 features have been evaluated. The resulting set of feature vectors has been subsequently used as a basis for constructing three- and four element feature spaces, to be used in classification of test utterances (Figure 4). All possible three- and four-dimensional feature vectors were considered as candidate feature spaces. However, not all of them were actually used in further analyses. Every subset was subject to the ‘feasibility’ test: if its components were ‘excessively’ correlated, they were considered to span a poor feature space. The maximum-correlation threshold was established based on statistical properties of input examples. Only subsets that passed the feasibility test were adopted for further emotion-classification procedures.

### 3.2. Emotion Classification

**Figure 5.**
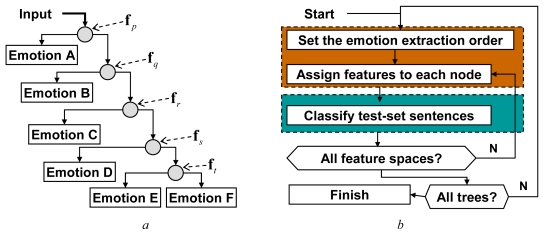
A structure of the adopted binary decision trees, where five different feature sets (**f***p*, **f***q*, **f***r*, **f***s*, **f***t*) are used for classification at each node (a); block diagram of the feature selection process, where the tree generation is highlighted in orange and classification—in cyan (b).

Emotion classification has been performed using binary decision trees, where one-to-many dichotomies are resolved at every node (Figure 5a). Each dichotomy is the problem of extracting a particular emotion from a mixture of the remaining components (the mixtures are tree-level dependent). *k*-nearest neighbor classification (*k*-NN), with *k* = 3, has been used for emotion extraction at consecutive tree-levels. Since six emotions have been subject to recognition, corresponding decision trees were composed of five levels. Each of the 120 possible tree structures, defined by different emotion extraction orders, has been examined, as any order could appear to give an optimal classifier.

A procedure that has been used for feature evaluation involves iterative repetition of the two steps: decision-tree generation and emotional speech classification (Figure 5b). The first step involves two operations. It begins with selection of a tree structure, *i.e.*, selection of the emotion-extraction order (e.g., ‘joy’ becomes the emotion A, B is ‘fear’ *etc.*). Next, either three- or four-element feature subsets that passed the feasibility test are assigned to each node of the tree. A node preset with some feature subset (e.g., a triplet *f_α_, f_β_, f_γ_*) becomes a 3-NN classifier, defined in a feature space spanned by the subset components (*f_α_, f_β_, f_γ_*) and preset with training set examples (as it can be seen in Figure 4).

Classification of a test-set utterance begins with an evaluation of features relevant for the tree's top-node (components of a vector **f***_p_*), followed by the first 3-NN classification. If the classification is inconclusive, subsequent decision levels are considered. Consecutive feature subsets (**f***q*, **f***r*, …) are evaluated and used for classification at subsequent nodes, until a leaf of the tree is reached. After all of the test-set sequences are processed, performance of the currently used tree is recorded, and the whole procedure is repeated for the new emotion-extraction order, until all possible tree structures are examined. The outcome of the procedure is a set of trees with top 5% recognition scores.

## 4. Experimental Evaluation of the Proposed Approach

Two different databases of emotional speech were used to evaluate the proposed approach. The first one [14], contains over five hundred sentences, uttered in German by five males and five females. The database includes seven different categories of emotional speech: “anger”, “fear”, “sadness”, “boredom”, “joy”, “disgust” as well as “no emotion”. The second database is a database of 240 sentences uttered in Polish [15]. Six types of emotions are expressed (“anger”, “fear”, “sadness”, “boredom”, “joy” and “no emotion”) by eight speakers (four males and four females). To establish a reference level for automatic emotion sensing, performance of human subjects has been evaluated for both databases (yielding over 80% for the German one and 72% for the Polish one). To enable cross-language comparisons of emotion sensing performance, we focused on classification of six emotions that are common to both databases.

There exist two basic experimental setups that can be used in emotion sensing: speaker-dependent recognition and speaker-independent recognition. In the former case, a classifier is trained and executed on samples taken from the same speaker, whereas in the second case, samples used for training and samples used for testing come from different speakers. Due to a limited size of the databases, which makes speaker-dependent recognition outcome statistically irrelevant, we decided to use another two categories of classification experiments: speaker-independent and speaker semi-independent recognition. In the former case, training and test sets were disjoint at the speaker's level, *i.e.*, decision trees were derived using sentences uttered by some speakers, whereas recognition was made based on sentences uttered by the remaining speakers. In the latter case, training and test sets are disjoint at the sentence level, *i.e.*, all utterances of all speakers are lumped together and some of them are used for training, whereas the remaining ones are used for testing. This latter setup is simpler (a classifier has a chance of learning some knowledge on all speakers), yet it is of clear practical use.

A total of sixteen sets of experiments have been performed, each featuring either of the two databases (Polish or German), either of the two recognition setups, either of the two considered feature space cardinalities (three- or four-element feature vectors) and either of the two genders. The last two categories were introduced due to gender-specific differences in vocal emotion-expression. Statistics of experimental material usage have been provided in Table 2. Due to the limited database size, three-fold cross-validation has been used during experimental evaluation of the presented procedure.

The main objectives of the experiments were to assess, whether the newly introduced descriptors can improve emotional speech recognition, to identify features that play a dominant role in discriminating particular emotions and to examine a language-context in emotion sensing.

### 4.1. Selection of the Feature-Subset Acceptance Threshold

Only feature subsets that pass the aforementioned feasibility test can be used for emotion classification. An exact threshold that determines rejection or acceptance of a given feature subset during the feasibility test has been established in the following way. First, four mean values: *μ_e_, μ_p_, μ_t_, μ_v_* were computed for pair-wise feature correlations within the considered four groups of descriptors (comprising energy, pitch, temporal and variability characteristics). We assumed the acceptance threshold *T* to be set at the level:

$$T=\min\mu_{e},\mu_{p},\mu_{t},\mu_{v}-\sigma$$

Adoption of the parameter *σ*, which is the minimum standard deviation computed over the four considered groups, enabled to substantially reduce (by approximately 98%) the number of candidate subsets for further testing, and thus, to speed-up the presented, computationally-costly procedure (however, still, approximately ten thousand triplets and over three hundred thousand quadruples remained for testing).

### 4.2. Descriptor Performance Evaluation

An outcome of emotion classification experiments is a set of the best-performing (top 5%) decision-trees. Depending on a particular experimental setup, the resulting sets comprised between sixteen elements (for feature-quad based, speaker semi-independent recognition performed for male-recordings from Polish-database) and forty-six elements (for triplet based speaker-independent recognition, male-recordings, German database). Since five feature vectors are assigned to each decision-tree, a total of 90-to-230 feature vectors were available for examination for the considered emotion classification variants.

**Figure 6.**
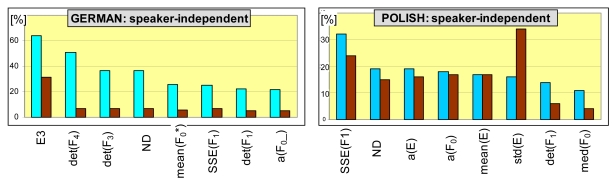
Best-performing features in emotion sensing for speaker-independent recognition among triplets (brown bars) and quadruples (turquoise bars) for German and Polish databases.

Histograms of features, which appear the most frequently among the winning trees and both databases, are shown in Figure 6. Characteristics that are most frequently appointed for feature spaces are: total energy of the frequency band that spans between 1.3F_0_ and 1.5F_0_, where F_0_ is an estimated pitch (denoted by E3), normalized duration of an utterance (ND) and Poincare map descriptors: determinants of co-variance matrices for the first, third and fourth formants (det(F1), det(F3), det(F43)), sum of squares due to error for the first formant (SSE(F1)), as well as linear regression coefficients of energy (a(E)) and pitch (a(F_0_)). Of other parameters, median pitch (med(F0), mean of local minima of pitch (F_0_*) and a linear regression slope for smoothed pitch (a(F0_)) can be also frequently found. One can observe that the proposed voiced-speech variability descriptors frequently appear among the feature spaces that produce the best emotion sensing rates (similar results have been observed for speaker semi-independent recognition). Also, their significance can be high: although for triplet-based recognition, energy-descriptors (sttd(E) and E3) are undisputed winners, first-formant variability is the most frequent member of the best performing feature spaces for quadruple-based recognition for Polish database.

**Figure 7.**
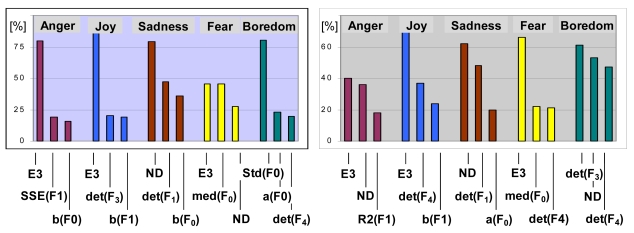
Best-performing features among triplets (a) and quadruples (b) in extracting particular emotions for German database, speaker-independent experiment.

The adopted classification method enables to analyze a role of different features in extracting particular emotions. Sample histograms of features that were most frequently used in specific emotion extraction, have been shown in Figure 7. Again, the proposed descriptors are frequent among the most discriminative feature spaces. The proposed group of emotional speech characteristics appears well-suited for modeling subtle processes that accompany basic emotion-expression mechanisms. For example, additional layer of muscle activity, can be noticeable for emotions with more ‘chaotic’ expression, associated with tremors, such as fear, or can be negligible for strongly developing emotional responses, like anger or joy. The corresponding jitter of vocal folds seems to be grasped by deviations from linear prediction of Poincare-map distributions (given e.g., by SSE) or by assessment of the coefficient of determination (R2) that reflects a level of dependence between consecutive acoustic events. Variability in several other mechanisms of speech production, such as breathing rate and intensity or tensions of vocal tract walls are likely to be expressed through total scatter of Poincare-map samples (evaluated using determinant or trace).

Emotional speech classification results are summarized in Table 3. Given a large, six-element set of target categories, the obtained rates can be considered very high for both speaker semi-independent and speaker-independent recognition (rates at the level of 80% were reported only for experiments involving two or three categories of emotions [11]). As it was expected, four-element feature sets provide a significant increase in recognition accuracy for both databases, as compared to feature-triplet based classification. This means that novel information is introduced by the fourth feature-vector component, and thus, it supports a hypothesis of existence of more than three, relatively uncorrelated groups of emotional speech characteristics.

To assess a role of voiced-speech short-term variability in emotion sensing, we decided to remove all of the newly proposed descriptors from the considered feature pool and to repeat the experiments. The results, summarized in Table 4, show a noticeable drop in classification performance, after removal of the proposed features. This clearly indicates both significance of the variability factor in emotion representation and suitability of the descriptors proposed in the paper for emotion sensing.

## 5. Conclusions

A novel family of voiced-speech pronunciation variability descriptors that has been proposed in the paper, proved to provide an important extension to a set of features commonly used for emotional speech characterization. The application of the proposed descriptors for emotion-classification yielded speaker-independent emotion sensing rates at the order of 80% for the six-category problem. It has been shown, that the proposed features significantly contribute to these high scores, and that they are frequently present among the most discriminative feature spaces.

The proposed concepts were verified using two databases of emotional speech, uttered in two different languages. Although a size of these databases does not allow to draw far-reaching conclusions (in fact, there exists no sufficiently extensive database of emotional speech), however, the observed tendencies are consistent and clearly identify the proposed descriptors as viable candidates for cross-language emotional speech representation.

The procedure adopted for verifying the proposed concepts—feature selection performed by means of binary decision-trees—was aimed at gaining the maximum insight into emotion discrimination capabilities of different speech-signal features. Therefore, a choice of some other emotional speech recognition strategy, such as for example Support Vector Machines, and use of the identified highly discriminative feature spaces, might result in even better recognition performance. Also, given the ‘enriched’ pool of initial descriptors of emotional speech, one can consider executing feature-extraction rather than feature-selection.

There exist several directions that can be considered for extending the reported research. For example, one of the interesting aspects of phone-uttering variability is its dynamics. Instead of focusing on Poincare-mapping results, one can focus on dynamics of the mapping process, for example, by building probabilistic models (e.g., HMM) of its evolution. This approach might bring yet another category of emotion-specific descriptors. Also, one can consider a variety of other properties of speech-signal to be used in modeling of emotion-specific jitter and use a variety of other approaches for extraction of emotion-specific information.

## Figures and Tables

**Table 1.** Correlation coefficients for selected characteristics of emotional speech for two emotional speech databases (all presented variability descriptors are derived for the first formant frequency—F1).

	**ND**	**MP**	**ME**
**det(F1)**	-0.08	-0.16	0.03
**SSE(F1)**	0.21	0.09	0.11
**R2(F1)**	-0.03	-0.27	-0.37

**Table 2.** A size of training and test sets used in emotion sensing.

	German Database		Polish Database	

	Speaker-independent	Speaker semi-independent	Speaker-independent	Speaker semi-independent
Training set	54^(*)^ / 54 up to 5 sentences per each emotion from 2 speakers	**60 / 60** (2 sentences per each emotion per each speaker)	**60 / 60** (5 sentences per each emotion from 2 speakers)	**48 / 48** (2 sentences per each emotion per each speaker)
Test set	**87 / 81** up to 5 sentences per each emotion from 3 speakers	**79 / 75** up to 3 sentences per each emotion per each speaker	**60 / 60** (5 sentences per each emotion from 2 speakers)	**72 / 72** (3 sentences per each emotion per each speaker)

(*) Female speakers / male speakers

**Table 3.** Emotion classification results with three- and four-element feature sets.

	**Speaker independent**		**Speaker semi-independent**	
	Triplets	Quads	Triplets	Quads
German database	72.2%	79.2%	76.3%	82.6%
Polish database	76.1%	81.9%	80.8%	85.5%

**Table 4.** Emotion sensing rates for two different initial pools of features: without the proposed variability descriptors (Basic) and with the descriptors (Extended), for four-element feature vectors.

	**Speaker independent**		**Speaker semi-independent**	
	Basic	Extended	Basic	Extended
German database	66.6%	79.2%	74.3%	82.6%
Polish database	64.4%	81.9%	76.8%	85.5%
